# Intrinsic and extrinsic drops in open-circuit voltage and conversion efficiency in solar cells with quantum dots embedded in host materials

**DOI:** 10.1038/s41598-018-30208-z

**Published:** 2018-08-03

**Authors:** Lin Zhu, Hidefumi Akiyama, Yoshihiko Kanemitsu

**Affiliations:** 1 Institute for Solar Energy Systems, Sun Yat-sen University, Guangzhou, 510006 China; 2 Institute for Solid State Physics, University of Tokyo, Kashiwa, Chiba 277-8581 Japan; 3 AIST-UTokyo OPERANDO-OIL, University of Tokyo, Kashiwa, Chiba 277-8589 Japan; 4 Institute for Chemical Research, Kyoto University, Uji, Kyoto 611-0011 Japan

## Abstract

We systematically analyzed the detailed-balance-limit-conversion efficiency of solar cells with quantum dots (QDs) embedded in host materials. We calculated their open-circuit voltage, short-circuit current, and conversion efficiency within single-photon absorption conditions, both in the radiative limit and in other cases with non-radiative recombination loss, using modeled absorption band with various absorptivities and energy widths formed below that of the host material. Our results quantitatively revealed the existence of intrinsic and significant drops in the open-circuit voltage and conversion efficiency of QD solar cells, in addition to extrinsic drops due to degraded material quality.

## Introduction

On the basis of solar cells incorporating quantum structures as wells (QWs) and dots (QDs), vast varieties of new concepts have been studied for improving conversion efficiency, such as increase in short-circuit current (*J*_*sc*_) via excitonic absorption, multi-exciton generation, and multi-photon-absorption, and as increase in open-circuit voltage (*V*_*oc*_) via reducing mismatch between absorption and emission solid angles^1–23^. One of the most intensively studied is a type of QD solar cells with QDs embedded in a bulk host material, which are intended to realize the concept of intermediate-band (IB) solar cells^1–15^. Empirically, however, *J*_*sc*_ of such solar cells has only been moderately improved, while the open-circuit voltage (*V*_*oc*_) has been lowered, and the conversion efficiency of those cells has been lowered compared with bulk host-material solar cells^4–9^, which should be quantitatively analyzed in comparison with theories. In this work, we focus here on this issue.

This type of QD solar cells and their experimental data have mostly been compared with IB-solar-cell model theories^1,2^. In those theories, a chemical potential or carrier population in an IB or QD states is isolated from that in the host material due to strong phonon-bottleneck effects, and only the latter is connected to the external voltage, which is the key to implement the concept of IB solar cells with minor voltage degradation. Therein, carrier extraction is prohibited after single-photon absorption to QD states, but needs two- or more-photon absorption processes, that is, carriers that have been pumped into QD levels should be pumped again by absorbing a second photon into conduction band^1,2^. On the other hand, experimentally measured *V*_*oc*_ and conversion efficiency of QD solar cells have been significantly lowered from bulk host-material solar cells in many cases^4–9^. The mechanism for these phenomena has to be investigated quantitatively or systematically. The growth of Stranski-Krastanov-mode self-assembled QDs may induce additional defects and/or dislocations due to strain accumulation, which may result in low material quality or radiative efficiency degrading *V*_*oc*_ and conversion efficiency. Thus, the experimentally observed low *V*_*oc*_ and conversion efficiency could be ascribed to the low material quality of QDs or host materials in QD solar cells. While researchers have continued their efforts to improve the growth of QDs^8–13^, improvements in *V*_*oc*_ and conversion efficiency are still difficult to implement compared to the case of bulk-material cells without QDs. To escape this stalemate, quantitative examination on the basis of fundamental and general theories is necessary, to analyze whether the voltage drop and resulting reduced conversion efficiency originate from an intrinsic mechanism or from extrinsically inferior material quality.

Shockley-Queisser (S-Q) detailed-balance-limit theory is best suited for this purpose^24,25^. This theory has the excellent quality of allowing the determination of the upper limit of conversion efficiency with only the absorption spectrum of a solar cell, regardless of its structural details. More recently, we developed an extended theory to incorporate the extrinsic effects of non-ideal material quality, as indicated by the internal radiative efficiency (*η*_*int*_) below unity^26–29^. The objective of this work is to use the extended detailed-balance-limit theory to quantitatively analyze the voltage drop and reduced conversion efficiency caused by intrinsic physics in QD solar cells, as apart from the contributions of extrinsically low material quality.

For QW solar cells, there has been an argument in the context of Shockley-Queisser detailed-balance theories that the conversion efficiency of QW solar cells cannot exceed that of a bulk cell with the optimum band gap, and that they are only useful in extending their band edges when no bulk material with the proper gap is found^30,31^. The same argument could be generally possible including the cases for QD and other quantum-structured solar cells, but no systematic or detailed study has been reported on this point.

**Figure 1:**
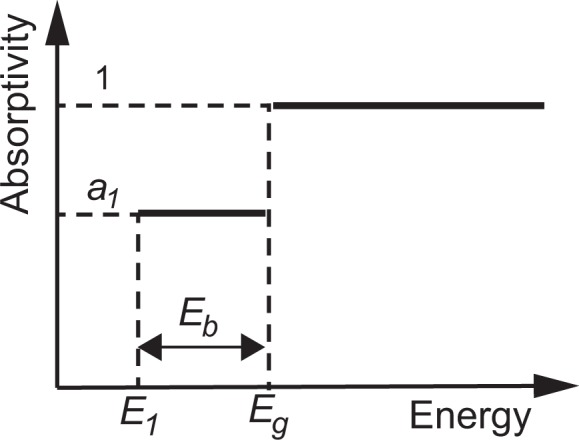
Modeled absorption spectrum of quantum-structural solar cells with a step-function tail below the host-material bandgap *E*_*g*_.

In this work, we model the absorption spectrum of a QD solar cell as a simple two-step function at the host-absorption region (absorptivity is set as 1 for photons with energy greater than bulk-material bandgap *E*_*g*_) and QD-absorption region (as a parameter *a*_1_ < 1 for photons with energy between *E*_*g*_ and the ground energy level arising from QD *E*_1_), demonstrated as Fig. 1 and formulated as Eq. (6), which is approximately comparable with reported experimental external quantum efficiency of QD solar cells^8,15^. Here all absorption processes are assumed as one-photon absorption, neglecting two- or multi-photon absorption processes. This model is used to evaluate the intrinsic and extrinsic upper limits of conversion efficiency with *J*_*sc*_ and *V*_*oc*_ for quantum structural solar cells on the basis of the extended detailed balance theory within one-photon-absorption processes. Though we hereafter denote QD as a representative case, this absorption-spectrum model is very general and applicable not only to QDs, but also to other quantum or nano structures such as wells, wires, disks, and rods. The results clarify that the introduction of low-density QDs (QWs, wires, etc.) causes a significant drop in *V*_*oc*_ with very a small gain in *J*_*sc*_, hence resulting in very low efficiency. As the density (number) of QDs is increased, *J*_*sc*_ is increased proportionally, and the efficiency rises accordingly. When the density (number) of QDs becomes sufficiently high, the conversion efficiency, *J*_*sc*_, and *V*_*oc*_ become equal to those of a bulk solar cell made of the QDs material with a low bandgap. Note that these are intrinsic and unavoidable effects stemming from the absorption spectrum of the QDs solar cells. Extrinsic low material quality further degrades *V*_*oc*_ and conversion efficiency.

## Results

We calculated the detailed-balance-limit value of conversion efficiency (*η*_*sc*_) as a function of *a*_1_ (the absorptivity arising from QD) between 0 and 1, which is as explained in the method section^28,29^. Considering a typical example of In_*x*_Ga_1−*x*_As QDs embedded in a GaAs host material, we assume *E*_*g*_ = 1.4 eV and the binding energy (*E*_*b*_ = *E*_*g*_ − *E*_1_) to be between 0.001 eV and 0.6 eV. In addition to the radiative-limit case with internal radiative efficiencies of host/QD material (${\eta }_{int}^{host/QD}=1$), calculations were also performed for various other ${\eta }_{int}^{host}$ and ${\eta }_{int}^{QD}$, down to 10^−5^.

**Figure 2:**
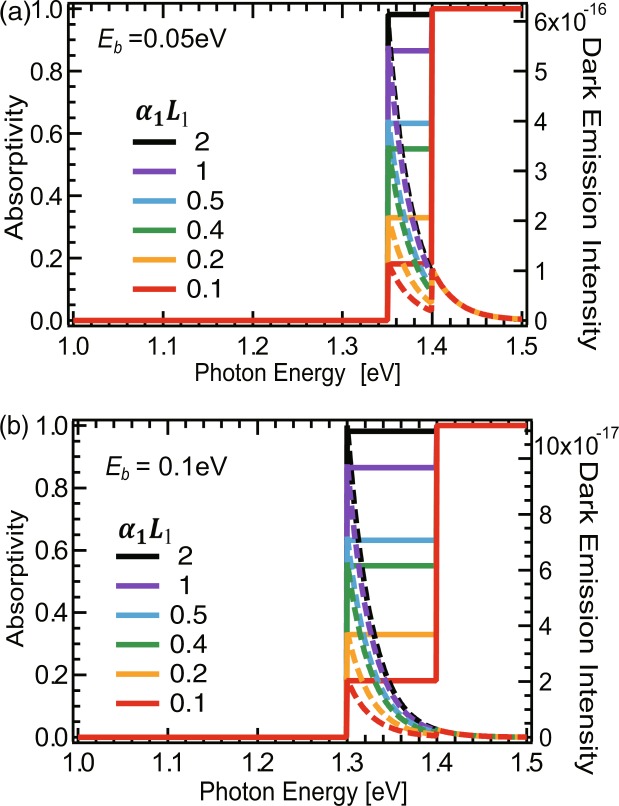
Absorptivity spectrum (solid) and dark emission spectrum (dashed) of QD solar cells with a host material *E*_*g*_ of 1.4 eV, binding energies of (**a**) *E*_*b*_ = 0.05 eV and (**b**) *E*_*b*_ = 0.1 eV, and various QD absorptivities *α*_1_*L*_1_.

Figure 2 shows examples of the absorptivity spectra and calculated dark emission spectra of QD solar cells for various values of absorptivity *a*_1_ and binding energy *E*_*b*_. In Fig. 2(a) with *E*_*b*_ = 0.05 eV, each dark emission spectrum clearly exhibits two peaks at *E*_*g*_ and *E*_1_, emitted from the host material and the QDs, respectively, whose intensities change with the values of *α*_1_*L*_1_. For large *a*_1_ (or large *α*_1_*L*_1_), the QD emission is dominant, whereas for moderate *α*_1_*L*_1_ around 0.1, both emissions from QD and host material are comparable, and for very small *α*_1_*L*_1_, the host material emission becomes dominant. For large binding energy *E*_*b*_ = 0.1 eV much greater than thermal energy *E*_*T*_ = *k*_*B*_*T* ≈ 0.026 eV at temperature *T* = 300 K, emission mostly comes from QDs rather than host material, as shown in Fig. 2(b).

**Figure 3:**
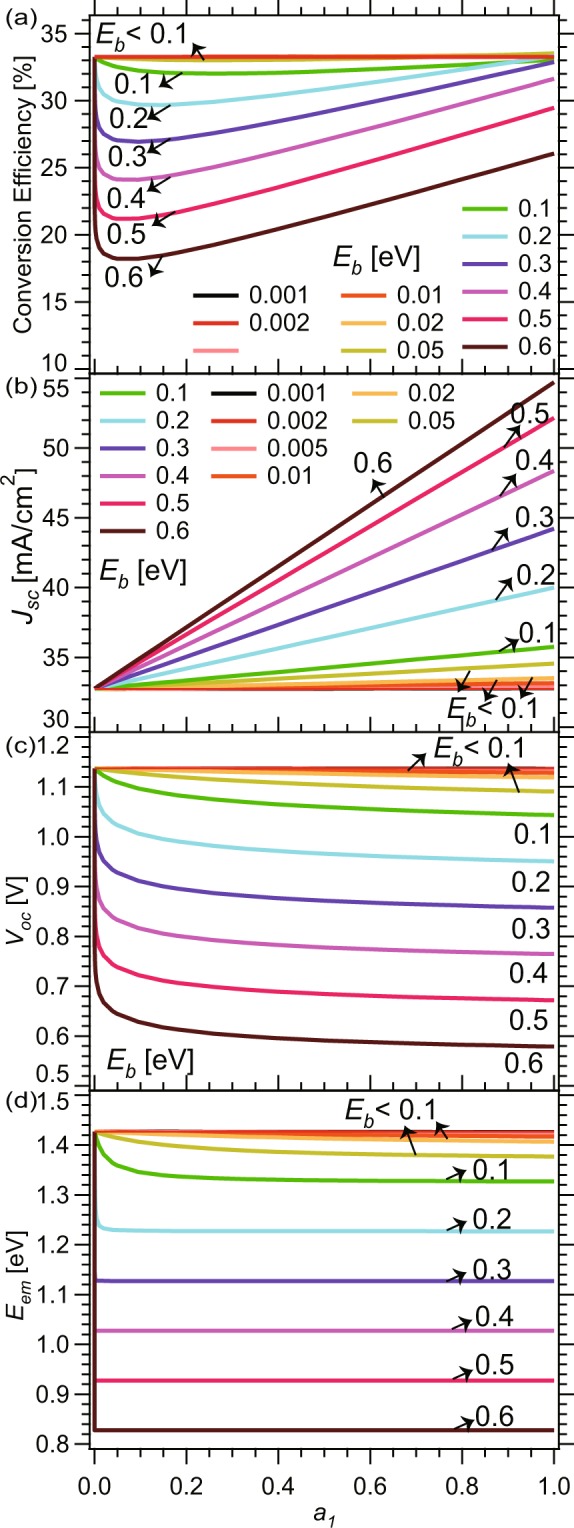
(**a**) Conversion efficiency, (**b**) *J*_*sc*_, (**c**) *V*_*oc*_, and (**d**) effective bandgap of QD solar cells with host material *E*_*g*_ = 1.4 eV and ideal internal radiative efficiency ${\eta }_{int}^{host/QD}=1$ for varied *a*_1_ and *E*_*b*_.

Figure 3(a) shows the calculated detailed-balance-limit-conversion efficiency, *η*_*sc*_, in the radiative limit (${\eta }_{int}^{host/QD}=1$). It converges to 33% at *a*_1_ = 0, corresponding to the efficiency limit of a bulk GaAs solar cell without QDs. It stays at 33% for various values of *a*_1_ if the QD binding energy, *E*_*b*_, is smaller than 0.1 eV. However, in cases where *E*_*b*_ is above 0.1 eV, as *a*_1_ increases from 0, the conversion efficiency first drops drastically from a value of 33%, and then increases almost linearly. At *a*_1_ = 1, the conversion efficiency is nothing but that of a bulk solar cell with a bandgap of *E*_1_ = *E*_*g*_ − *E*_*b*_. To clarify the origins of the behaviors of *η*_*sc*_ presented in Fig. 3(a), we next calculated the corresponding *J*_*sc*_ and *V*_*oc*_, as shown in Fig. 3(b,c).

In Fig. 3(b,c), *J*_*sc*_ and *V*_*oc*_ are 32.7 mA/cm^2^ and 1.14 V, respectively, at *a*_1_ = 0 or when *E*_*b*_ < 0.1 eV, that is, the same values exhibited by a bulk GaAs solar cell with *E*_*g*_ = 1.4 eV and ${\eta }_{int}^{host/QD}=1$. At *a*_1_ = 1, they are identical to those of a bulk solar cell with a bandgap of *E*_1_. In Fig. 3(b), the boost of *J*_*sc*_ increases linearly as *a*_1_ increases, where the slopes increase as *E*_*b*_ increases, caused from the sub-band absorption add-on. For these cells only being introduced a few narrow-bandgap materials with very tiny *a*_1_, *J*_*sc*_ add-on is negligible, almost pinned at 32.7 mA/cm^2^, regardless of how much *E*_*b*_ is. On the other hand, as *a*_1_ increases in Fig. 3(c), *V*_*oc*_ drops very drastically and steeply near *a*_1_ = 0, flattens at *a*_1_ below 0.1, and then converges to the values at *a*_1_ = 1. It is evident that the conversion efficiency is almost proportional to the product of *J*_*sc*_ and *V*_*oc*_. In fact, we confirmed that fill factors do not change much in the present parameter regions^24^.

The significant inherent drop in *V*_*oc*_ near *a*_1_ = 0 in Fig. 3(c) can be interpreted, via expression of *V*_*oc*_;

1 $$\begin{array}{rcl}{V}_{oc}({a}_{1},{E}_{1},{E}_{g}) & = & {V}_{T}\,\mathrm{ln}({J}_{sc}/{J}_{0})\\  & = & {V}_{T}\,\mathrm{ln}\,{J}_{sc}-{V}_{T}\,\mathrm{ln}\,q({R}_{ext0}^{host}+{R}_{ext0}^{QD})\\  & = & {V}_{T}\,\mathrm{ln}\,{J}_{sc}-{V}_{T}\,\mathrm{ln}\,q{R}_{ext0}^{host}-{V}_{T}\,\mathrm{ln}(1+\frac{{R}_{ext0}^{QD}}{{R}_{ext0}^{host}})\end{array}$$

derived from Eqs (8–11) with ${\eta }_{int}^{host/QD}=1$, where *V*_*T*_ = *k*_*B*_*T*/*q* ≈ 0.026 V is the thermal voltage, and ${R}_{ext0}^{host}$ and ${R}_{ext0}^{QD}$ are the radiative recombination flux from host material and QD under dark condition, at *T* = 300 K. Here, dependence of *V*_*oc*_ on *a*_1_ via *J*_*sc*_ is small, and that via the changes of the dark current (*J*_0_) is dominant. Then, for ${E}_{b}\gg {E}_{T}$(=*V*_*T*_*q*), Eq. (1) becomes approximately as

2 $${V}_{oc}({a}_{1},{E}_{1},{E}_{g})={V}_{oc}^{Bulk}({E}_{g})-{V}_{T}\,\mathrm{ln}(1+{a}_{1}\frac{{E}_{1}^{2}}{{E}_{g}^{2}}\,\exp \,\frac{{E}_{b}}{{E}_{T}})$$

At *a*_1_ = 0, *V*_*oc*_ is the value of open-circuit voltage ${V}_{oc}^{Bulk}({E}_{g})$ for host-material-bulk cells with *E*_*g*_. As *a*_1_ is increased from 0, *V*_*oc*_ goes down steeply, because coefficient exp(*E*_*b*_/*E*_*T*_) of *a*_1_ in the second term in Eq. (2) is very large, because of ${E}_{b}\gg {E}_{T}$.

For $\exp ({E}_{b}/{E}_{T})\gg 1/{a}_{1}$, Eq. (2) is expressed approximately as,

3 $${V}_{oc}({a}_{1},{E}_{1},{E}_{g})={V}_{oc}^{Bulk}({E}_{1})-{V}_{T}\,\mathrm{ln}\,{a}_{1}$$

Here, the contribution of *V*_*T*_ ln *a*_1_ is smaller than 60 mV for 0.1 < *a*_1_ < 1, and thus *V*_*oc*_ is close to open-circuit voltage ${V}_{oc}^{Bulk}({E}_{1})$ for bulk cells with *E*_1_. This arises from that *J*_0_ in cells with low *E*_1_ for large *E*_*b*_ is almost governed by the recombination current arising from QD $(q{R}_{ext}^{QD})$, with band edge at *E*_1_ and absorptivity *a*_1_. Crudely speaking, the more *a*_1_ and the lower *E*_1_ indicate the more radiative emission losses from QD and further more dark current, which lowered *V*_*oc*_.

Figure 3(d) plots the emission energy, *E*_*em*_, defined as the center-of-mass energy in the emission spectra. For a large binding energy of *E*_*b*_ > 0.1 eV, *E*_*em*_ immediately drops to *E*_1_ as *a*_1_ increases from 0. For shallow bonding energy *E*_*b*_ < 0.1 eV, *E*_*em*_ drops only slightly and more gradually and stays close to the host bandgap, *E*_*g*_. The behaviors of *E*_*em*_ represent the changes in their emission spectra in Fig. 2(a,b), where the position of the dominant emission peak is switched from *E*_*g*_ to *E*_1_ as *a*_1_ and *E*_*b*_ increases. In the detailed-balance-limit theory with a radiative limit (${\eta }_{int}^{host/QD}=1$), carrier loss only occurs via radiative emission, which is determined by the product of absorptivity *a*(*E*_*em*_) and the 300-K blackbody emission intensity, *B*(*E*_*em*_), at the emission energy *E*_*em*_. Therefore, *E*_*em*_ can be interpreted as the effective band-gap energy determining *V*_*oc*_, corresponding to the first term in Eq. (3) mentioned above. This explains why *V*_*oc*_ drops steeply as *a*_1_ increases from 0, and its feature versus *E*_1_ at moderate and large *a*_1_ in Fig. 3(c) are similar to those of *E*_*em*_ in Fig. 3(d).

Note that the results in Figs 2 and 3 are all obtained in the radiative limit with ${\eta }_{int}^{host/QD}=1$. Therefore, these significant drops in the open-circuit voltage and conversion efficiency are intrinsic and unavoidable consequence of the absorptivity spectra of QD solar cells modeled in Fig. 1.

**Figure 4:**
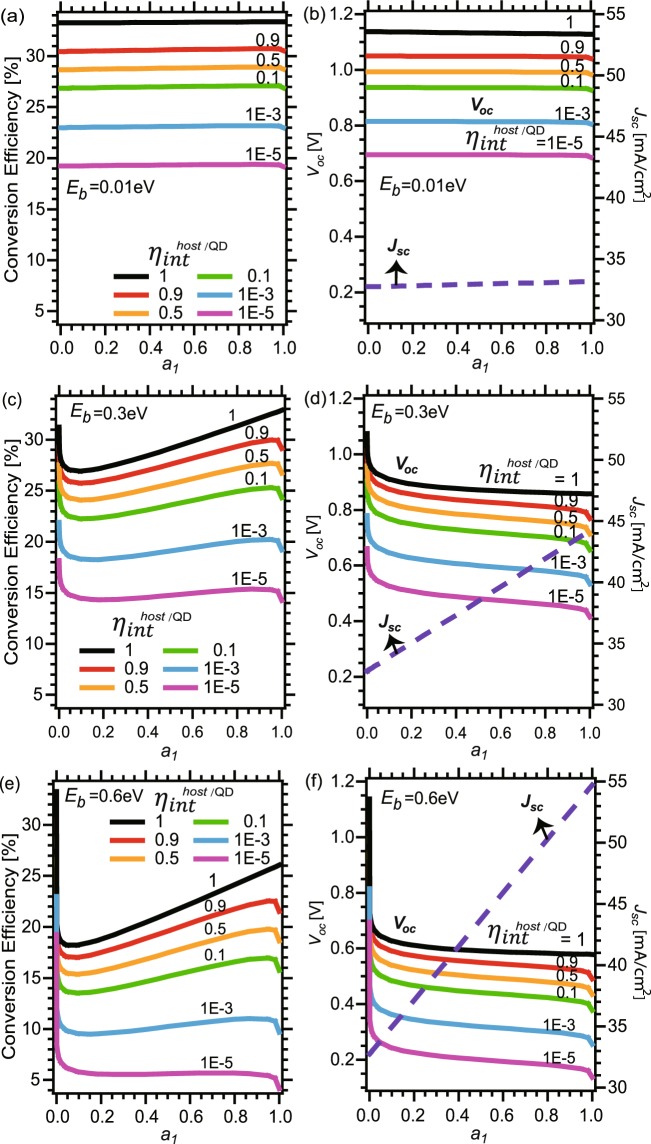
(**a**,**c**,**e**) Conversion efficiency, (**b**,**d**,**f**) *J*_*sc*_ (dashed) and *V*_*oc*_ (solid) of QD solar cells with host material *E*_*g*_ = 1.4 eV for varied *a*_1_ and ${\eta }_{int}^{host/QD}$ at *E*_*b*_ = 0.01 eV, 0.3 eV, and 0.6 eV.

Figure 4 exhibits the conversion efficiency (a, c, e), *J*_*sc*_ and *V*_*oc*_ values (b, d, f) of QD solar cells with varied material qualities arising from extrinsic origins or internal radiative efficiencies, *η*_*int*_, below 1 down to 10^−5^. Here, we assumed QD and host materials have the same *η*_*int*_. Three typical values are assumed for the binding energy *E*_*b*_, namely 0.01 eV (a, b), 0.3 eV (c, d), and 0.6 eV (e, f). A black curve in each panel represents data in the radiative limit (*η*_*int*_ = 1) without non-radiative recombination losses. We note that a slight drop of *η*_*int*_ from 1 to 0.9 already causes a drastic downward shift in conversion efficiency by about 2~3% absolute and in *V*_*oc*_ by 0.05~0.1 V. Significant drops in conversion efficiency and *V*_*oc*_ also occur as *η*_*int*_ degrades from 1 to 0.1, in this case by about 5~10% absolute and about 0.2 V, respectively. Further drops in conversion efficiency and *V*_*oc*_ with degradation of *η*_*int*_ by two orders of magnitude from 0.1 (or 10^−3^) to 10^−3^ (or 10^−5^) are by about 4~6% absolute and about 0.1~0.15 V, respectively. In addition, extrinsic drops slightly increase as *a*_1_ increases. Especially at *a*_1_ close to 1, conversion efficiency and *V*_*oc*_ for non-unity *η*_*int*_ steeply drop to the corresponding values for bulk cells with bandgap *E*_1_, notably differing from the gradual black curves of *η*_*int*_ = 1. This arises from a sharply increasing non-radiative recombination losses in cells with non-unity *η*_*int*_, when *a*_1_ approaching to 1. In this case, *α*_1_*L*_1_ becomes extremely large, which makes *η*_*ext*_ sharply reduced in Eq. (12) for *η*_*int*_ < 1, and causes the drops of *V*_*oc*_ and conversion efficiency.

**Figure 5:**
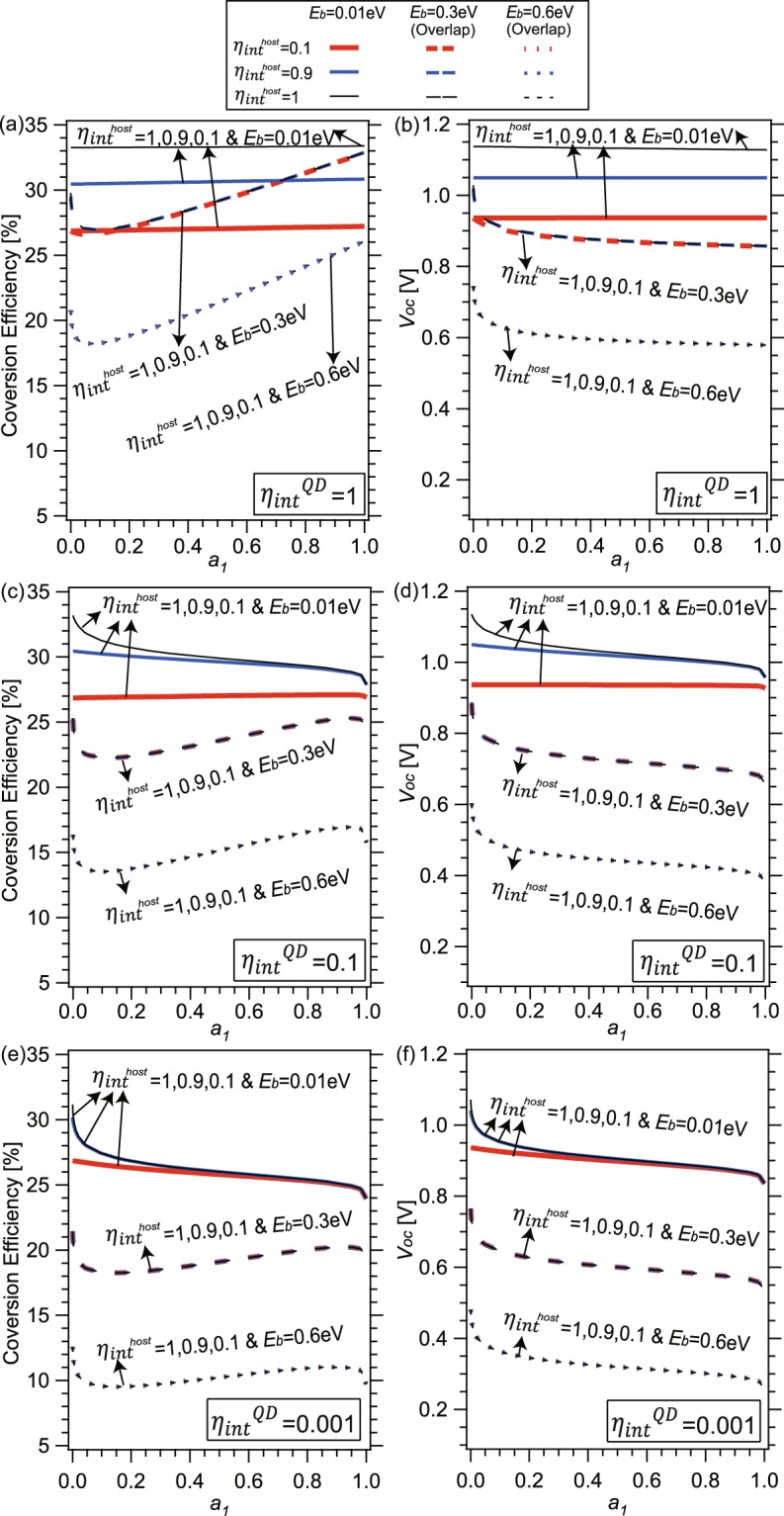
(**a**,**c**,**e**) Conversion efficiency, (**b**,**d**,**f**) and *V*_*oc*_ of QD solar cells with host material *E*_*g*_ = 1.4 eV for varied *a*_1_ and ${\eta }_{int}^{QD}$ at *E*_*b*_ = 0.01 eV, 0.3 eV, and 0.6 eV and ${\eta }_{int}^{host}=1$, 0.9, and 0.1.

Figure 5 exhibit the cell behaviors whose QD and host material qualities are different: three typical values are assumed for ${\eta }_{int}^{QD}$, namely 1 (a, b), 0.1 (c, d), and 0.0001 (e, f), and in each subplot ${\eta }_{int}^{host}$ are set as 1, 0.9, 0.1 and *E*_*b*_ also taken by 0.01, 0.3, 0.6 eV, respectively. Although the drop tendencies are similar to those with the same *E*_*b*_ in Fig. 4, they also reveal some different behaviors. For large *E*_*b*_ of 0.3 and 0.6 eV, *V*_*oc*_ and conversion efficiency for different ${\eta }_{int}^{host}$ are almost overlapping and only determined by *a*_1_, *E*_1_ and ${\eta }_{int}^{QD}$, because the behaviors of such cells with deep *E*_*b*_ greater than several *E*_*T*_ are very similar as those of bulk cells with bandgap *E*_1_. These are reasonable, because *V*_*oc*_ for large *E*_*b*_ is approximately expressed as

4 $$\begin{array}{rcl}{V}_{oc} & \approx  & {V}_{oc}^{radiative}+{V}_{T}\,\mathrm{ln}\,{\eta }_{ext}^{QD}\\  & = & {V}_{oc}^{radiative}-{V}_{T}\,\mathrm{ln}\,[1+\frac{4{n}^{2}{\alpha }_{1}{L}_{1}}{{a}_{1}}(\frac{1}{{\eta }_{{int}}^{QD}}-1)],\end{array}$$

which is independent of the host material quality ${\eta }_{int}^{host}$. Note that the 1st and 2nd terms in Eq. (4) serve as intrinsic and extrinsic drop, respectively.

In contrast, for cells with shallow *E*_*b*_, *V*_*oc*_ is determined both of host and narrow-gap materials. For low *a*_1_, where recombination current in host band dominates the dark current, the conversion efficiency and *V*_*oc*_ almost converge to the values of bulk cells with *E*_*g*_ and ${\eta }_{int}^{host}$, and are weakly dependent on *a*_1_ and ${\eta }_{int}^{QD}$. However, in the large *a*_1_ region where the recombination in QD becomes dominant, *V*_*oc*_ drops primarily by *E*_1_ and ${\eta }_{int}^{QD}$. Note here these colored curves in Figs 4 and 5 include both intrinsic and extrinsic drops in conversion efficiency and *V*_*oc*_, which are comparable with realistic data.

## Discussion

The above essential features of intrinsic and extrinsic drops in conversion efficiency and *V*_*oc*_ hold not only for the simple two-step-function model, but also for a finite-band-width model, where absorption band of QDs with absorptivity *a*_1_ starts at *E*_1_ and ends at *E*_2_(<*E*_*g*_) so that absorptivity gap exists between *E*_2_ and *E*_*g*_^15,30,32^. For example, we analyzed a case with *E*_2_ = *E*_1_ + 3*E*_*T*_, and found that *V*_*oc*_ stays almost the same as Fig. 3(c) obtained for the simple two-step-function absorptivity model, though *J*_*sc*_ is decreased due to the absorptivity gap between *E*_2_ and *E*_*g*_, and conversion efficiency is lowered accordingly. In short, the intrinsic *V*_*oc*_ drops are not sensitive to sub-band absorption profiles, but are sensitive to the energy position of the lowest absorption edge and the absorptivity amplitude at the edge. Indeed, this conclusion is also consistent with reports on the detailed-balance-limit efficiency for semiconductors with inhomogeneous alloy broadening with Gaussian tails^33–35^ or with excitonic band-edge peaks^32^. It is of course consistent with previous reports on the detailed-balance-limit efficiency of QW solar cells^30^. It is interesting to examine our presented conversion-efficiency results at various *a*_1_ and *E*_*b*_ with the well-known ultimate efficiency as in S-Q paper^24^, that is, detailed-balance limit for the same two-step-function-absorption cells with temperature of 0 K under 6000 K-blackbody sun, expressed as,

5 $$\begin{array}{c}u({x}_{1},{x}_{g},{a}_{1})=\{\begin{array}{cc}\frac{{x}_{g}\,{\int }_{{x}_{g}}^{{\rm{\infty }}}\,{x}^{2}dx/({e}^{x}-1)}{{\int }_{0}^{{\rm{\infty }}}\,{x}^{3}dx/({e}^{x}-1)}, & {\rm{i}}{\rm{f}}\,{a}_{1}=0\,\\ \frac{{a}_{1}{x}_{1}\,{\int }_{{x}_{1}}^{{x}_{g}}\,\frac{{x}^{2}dx}{{e}^{x}-1}+{x}_{1}\,{\int }_{{x}_{g}}^{{\rm{\infty }}}\,\frac{{x}^{2}dx}{{e}^{x}-1}}{{\int }_{0}^{{\rm{\infty }}}\,{x}^{3}dx/({e}^{x}-1)}. & {\rm{i}}{\rm{f}}\,{a}_{1}\ne 0\end{array}\end{array}$$

**Figure 6:**
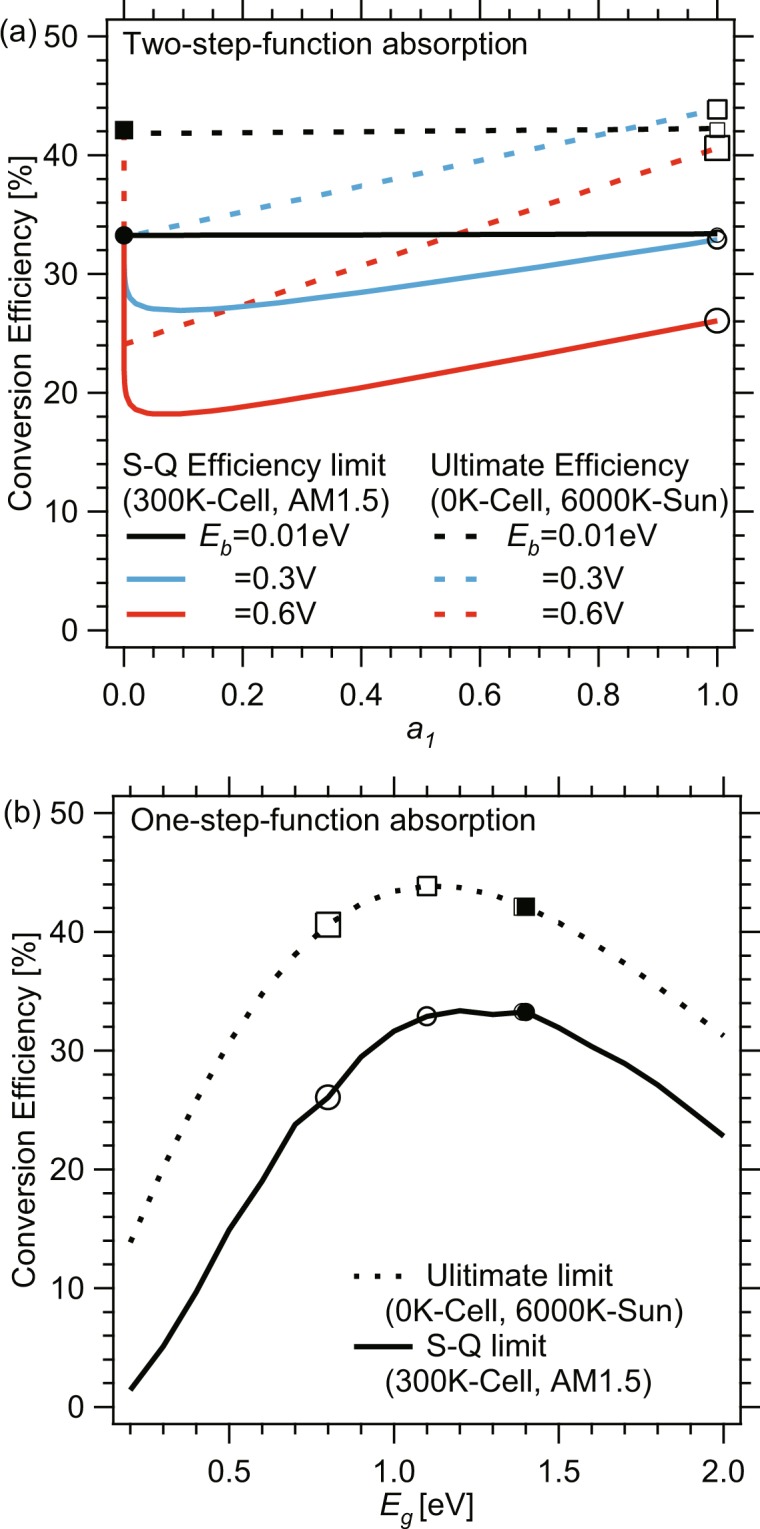
Comparison of ultimate efficiency of QD solar cells at 0 K with the efficiency of 300 K cells under AM1.5 for varied *a*_1_ and *E*_*b*_ = 0.01 eV, 0.3 eV, and 0.6 eV.

We plot, in Fig. 6(a), the ultimate efficiency by dashed lines, in comparison with our results in Fig. 3(a), for the three cases of *E*_*b*_ = 0.01, 0.3, and 0.6 eV (*E*_1_ = *E*_*g*_ − *E*_*b*_ = 1.39 eV, 1.1 eV, and 0.8 eV). We marked the two-limit points of *a*_1_ = 0 (filled symbols) and *a*_1_ = 1 (open symbols) for the ultimate efficiency (squares) and our present results (circles), which should be equal to the results of S-Q paper^24^. Figure 6(b) shows the ultimate efficiency and S-Q-limit efficiency for conventional single-step-function absorption model^24^, and indeed, the marked data points at *E*_*g*_ = 1.4 eV, 1.39 eV, 1.1 eV, and 0.8 eV are consistent with those marked by the corresponding symbols in Fig. 6(a).

Note in Fig. 6(a), that the intrinsic drop also occurs in ultimate efficiency at 0 < *a*_1_ < 1 brought by the narrow-gap materials introduction, similarly to our present results of QD cells at 300 K. At 0 K, all carriers relax down to the lowest possible energy levels of *E*_1_ and the recombination from QD confined level is the only recombination mechanism, which cause *V*_*oc*_ drop in the ultimate efficiency limit going down to *E*_1_/*q*. However, at a finite temperature *T*, typically for 300 K assumed in this work, all carriers generated from absorbed photons reach a Boltzmann distribution at *T*, and, as a result, *V*_*oc*_ drops down to below *E*_1_/*q*, resulting in our present efficiency limit curves being softer than those of ultimate limit.

It is important to emphasize that the detailed-balance-limit study with the two-step-function model is applicable not only for QW solar cells, but also for QD and other quantum-structure solar cells. Whether or not theoretical models and assumptions are correct for each case of experiments should be judged by examining agreements between experimental and theoretical results. Our present work provides systematic theoretical results for all of conversion efficiency, *V*_*oc*_, *J*_*sc*_, and emission photon energy *E*_*em*_ for such comparison.

One of the major and long lasting questions in solar-cell study is how close practical QD solar cells with embedded QDs in host materials are to the concept of IB solar cells^3,4,8,13^. The IB-solar-cell theoretical model^1,2^ assumes the phonon bottleneck effect, which was theoretically predicted for QDs having a discrete energy levels^36,37^: As the level spacings in QDs become larger, calculated carrier relaxation rates in QDs mediated by single phonon emission become slower due to energy and momentum conservation. This effect has been controversial, and consensus has not been established yet. In the presence of the phonon bottleneck effect, populations, and hence chemical potentials, of electrons and holes in the host material are isolated from those in an IB or QD states. Therefore, incorporation of IB or QD states in the middle of a pn-junction should cause only a small intrinsic *V*_*oc*_ drop. Moreover, single photon absorption in QDs cannot contribute to photo-current, but two- or multi-photon absorption processes are necessary to generate photo-current. The concept of IB solar cells is based on these grounds. On the other hand, our present detailed-balance calculations assume that the phonon bottleneck effect is absent or negligible. Then, the thermalization of conduction-band electrons across the host material and QDs is much faster than electron-hole recombination. In other words, the chemical potentials of conduction-band electrons are equal between in the host material and in the QDs. The same things are true for valence-band holes. Therefore, the intrinsic drop in *V*_*oc*_ occurs, and photo-current is induced via one-photon absorption processes to both of QDs and the host material.

Note that the IB-solar-cell model and our present detailed-balance model respectively deal with the opposite limits of slow (no) versus fast (instantaneous) carrier thermalization across the host material and QDs, assuming existence versus absence of the phonon bottleneck effect for carrier relaxation in QDs. It should be important to compare experimental data of QD solar cells with calculations via various possible models in the equal footing.

We point out that experimental characteristics of QD solar cells reported so far mostly showing degraded *V*_*oc*_ from those of reference-bulk solar cells^4–9^ may be explained as intrinsic by our present calculations, if fast carrier thermalization across the host material and QDs is the case. We note that this can be checked via electroluminescence or photoluminescence experiments by excitation into the host materials, because it predicts whether or not the luminescence intensities of QDs and a host-material follow the same Boltzmann distribution with the identical chemical potential and temperature, as shown in Fig. 2.

The efficiency calculated here on the basis of detailed-balance-limit theory under single-photon absorption only showed the case where embedded QDs bring down the performance of the host-material bulk cells. However, as we mentioned at the beginning of the introduction section, vast varieties of new concepts have been proposed for QD solar cells, and significant increase in short-circuit current, for example, via multi-photon absorption and multi-exciton generation, may compensate the voltage drops and result in overall improvement in conversion efficiency. Our present calculation results should be very important to evaluate such a break-even point for the new-concept QD solar cells, or to quantitatively analyze extrinsic and intrinsic drops in experimental conversion efficiency of fabricated samples of QD solar cells. In this sense, this work should be practically helpful in developing all of the new-concept QD solar cells.

## Conclusion

In summary, we have presented an analysis of the detailed-balance-limit conversion efficiency, short-circuit current, open-circuit voltage, and emission energy of QD solar cells with various parameters for the QD-absorption band below the host-material band gap. When the QD-absorption band has absorptivity, *a*_1_, of almost 0 or small QD-binding energy *E*_*b*_ below 0.1 eV, the cell is almost identical to a bulk-host-material solar cell. As *a*_1_ increases from 0 with *E*_*b*_ > 0.1 eV, *J*_*sc*_ increases linearly while *V*_*oc*_ drops steeply near *a*_1_ = 0 and becomes flat for *a*_1_ > 0.1. Nearly proportionally to the product of *J*_*sc*_ and *V*_*oc*_, the conversion efficiency drops sharply near *a*_1_ = 0 and then increases almost linearly. The center-of-mass emission energy, *E*_*em*_, plays the role of effective band-gap energy to determine *V*_*oc*_. Additional drops in conversion efficiency and *V*_*oc*_ occur with extrinsic degradation of material quality or internal radiative efficiency, *η*_*int*_. Our results suggest that drops of *V*_*oc*_ and conversion efficiency in QD solar cells may be caused by these intrinsic reasons as a result of fast carrier thermalization across the host material and QDs.

## Methods

Figure 1 shows the simple two-step-function absorptivity (*a*), related to the product of absorption coefficient (*α*) and material thickness (*L*), as *a* = 1 − exp(−2*αL*), which are given respectively by

6 $$\begin{array}{c}a=\{\begin{array}{cc}1, & {\rm{i}}{\rm{f}}\,E\geqslant {E}_{g}\\ {a}_{1}, & {\rm{i}}{\rm{f}}\,{E}_{g} > E\geqslant {E}_{1}\\ 0, & {\rm{i}}{\rm{f}}\,E < {E}_{1}\end{array}\end{array}$$

and

7 $$\begin{array}{c}\alpha L=\{\begin{array}{cc}5, & {\rm{i}}{\rm{f}}\,E\geqslant {E}_{g}\\ {\alpha }_{1}{L}_{1}, & {\rm{i}}{\rm{f}}\,{E}_{g} > E\geqslant {E}_{1}\\ 0, & {\rm{i}}{\rm{f}}\,E < {E}_{1}\end{array}\end{array}$$

for the absorption spectrum of a QD solar cell. Emphasize again, though we denote QD as a representative case in this paper, this absorption-spectrum model and conclusion are applicable not only to QDs, but also to other quantum or nano structures. Also all absorption processes are considered as one-photon absorption, rather than the two- or multi-photon absorption. In Eqs (6 and 7), the following assumptions are also made: the host material with bandgap *E*_*g*_ is thick enough to have an above-*E*_*g*_ absorptivity of nearly unity, while QDs with binding energies of *E*_*b*_ extend the low-energy absorption-band edge to *E*_1_ = *E*_*g*_ − *E*_*b*_ with an absorptivity of *a*_1_. Effects of more limited absorption band width of QDs are discussed in the discussion part of this paper. To investigate the upper-limit efficiency, it is also assumed that the solar cell has a perfect rear mirror to enable double-pass absorption. *αL* is taken to be greater than 5 above *E*_*g*_, while to be various values represented by parameter *α*_1_*L*_1_ for energies between *E*_*g*_ and *E*_1_. The density and absorption-oscillator strength of the QDs determine *α*_1_*L*_1_.

Once the absorptivity spectrum *a*(*E*) is given, the Kirchhoff law of radiation or the detailed-balance principle between photon absorption and emission with the Planck’s radiation formula for 300-K blackbody emission provides the dark emission spectrum of the solar cell at 300 K^25^. Under a Boltzmann-statistics approximation, the emission spectrum under bias voltage *V* is equal to the product of the dark emission spectrum and a voltage factor of exp(*V*/*V*_*T*_), where *V*_*T*_ = *k*_*B*_*T*/*q* ≈ 0.026 V is the thermal voltage at *T* = 300 K. Here we make an implicit assumption that photo-generated electrons and holes are in respective thermal equilibrium with the same carrier temperature of 300 K but with separated respective chemical potentials. Additionally, infinite carrier mobility, such that the difference in the chemical potentials of electrons and holes is uniform over the p-n junction and equal to the product of bias voltage and electron charge *qV*, is assumed in this model. This assumption is not realistic, but ideal, whose use is justified when evaluating the ideal theoretical upper limit of conversion efficiency. The Boltzmann-statistics approximation is known to cause deviation from rigorous Fermi-Dirac statistics for strongly concentrated illuminations, deep QDs, or strongly doped QDs, so we checked that the deviation is negligibly small for undoped QDs with the parameter regions covered in this paper.

By the carrier balance in a solar cell, the current *J* flowing out from the cell is equal to the difference between the photocurrent *J*_*sc*_, and the recombination-loss current arising from host and QD materials. Thus, the I-V characteristics are given by

8 $$J={J}_{sc}-q(\frac{{R}_{ext}^{host}}{{\eta }_{ext}^{host}}+\frac{{R}_{ext}^{QD}}{{\eta }_{ext}^{QD}})={J}_{sc}-q(\frac{{R}_{ext0}^{host}\,\exp \,\frac{V}{{V}_{T}}}{{\eta }_{ext}^{host}}+\frac{{R}_{ext0}^{QD}\,\exp \,\frac{V}{{V}_{T}}}{{\eta }_{ext}^{QD}})$$

9 $${J}_{sc}=q\,{\int }_{0}^{\infty }\,a(E)S(E)dE$$

10 $${R}_{ext0}^{host}=\pi \,{\int }_{{E}_{g}}^{\infty }\,a(E)B(E)dE$$

11 $${R}_{ext0}^{QD}=\pi \,{\int }_{{E}_{1}}^{{E}_{g}}\,a(E)B(E)dE$$

where *S*(*E*) and *B*(*E*) are solar and 300-K blackbody spectra^24^, respectively. In this paper, we use the solar spectrum of AM1.5 G with an incident power per unit area of *P*_*in*_ = 100 mW/cm^2^, and take only one-photon-absorption processes into account. The current-loss term $q{R}_{ext}^{host}/{\eta }_{ext}^{host}+q{R}_{ext}^{QD}/{\eta }_{ext}^{QD}$ in Eq. (8) includes radiative-recombination current loss from host- and QD-materials for external emission loss via front surface ($q{R}_{ext}^{host}+q{R}_{ext}^{QD}$) and non-radiative-recombination current loss (indicted by ${\eta }_{ext}^{host}$ and ${\eta }_{ext}^{QD}$). $q{R}_{ext0}^{host}$ and $q{R}_{ext0}^{QD}$ respectively represent the radiative-recombination current loss via the front surface in the dark. We apply the relation between external and internal radiative efficiency (*η*_*ext*_ and *η*_*int*_)^28,29^, as,

12 $$\frac{1}{{\eta }_{ext}^{host/QD}}-1=\frac{4{n}^{2}\overline{{\alpha }^{host/QD}}L}{\overline{{a}^{host/QD}}}(\frac{1}{{\eta }_{{int}}^{host/QD}}-1)$$

13 $$\overline{{a}^{host}}={\int }_{{E}_{g}}^{\infty }\,a(E)B(E)dE/{\int }_{{E}_{g}}^{\infty }\,B(E)dE$$

14 $$\overline{{a}^{QD}}={\int }_{{E}_{1}}^{{E}_{g}}\,a(E)B(E)dE/{\int }_{{E}_{1}}^{{E}_{g}}\,B(E)dE$$

15 $$\overline{{\alpha }^{host}}={\int }_{{E}_{g}}^{\infty }\,\alpha (E)B(E)dE/{\int }_{{E}_{g}}^{\infty }\,B(E)dE$$

16 $$\overline{{\alpha }^{QD}}={\int }_{{E}_{1}}^{{E}_{g}}\,\alpha (E)B(E)dE/{\int }_{{E}_{1}}^{{E}_{g}}\,B(E)dE$$

to introduce internal radiative efficiency $({\eta }_{int}^{host/QD})$ as pure indicators of the host/QD material quality of single-junction QD solar cells, separately from the cell geometry. Here, *n* and *L* are the reflective index and material thickness. $\bar{<mml:mpadded xmlns:xlink="http://www.w3.org/1999/xlink" lspace="-1.5pt">{a}^{host/QD}</mml:mpadded>}$ and $\bar{<mml:mpadded xmlns:xlink="http://www.w3.org/1999/xlink" lspace="-1.5pt">{a}^{host/QD}</mml:mpadded>}$ are the corresponding energy-averaged absorptivity and absorption coefficient for host/QD materials, respectively.
